# Glutamate-Evoked Ca^2+^ Responses in the Rat Suprachiasmatic Nucleus: Involvement of Na^+^/K^+^-ATPase and Na^+^/Ca^2+^-Exchanger

**DOI:** 10.3390/ijms24076444

**Published:** 2023-03-29

**Authors:** Pi-Cheng Cheng, Ruo-Ciao Cheng, Rong-Chi Huang

**Affiliations:** 1 Department of Physiology and Pharmacology, College of Medicine, Chang Gung University, Taoyuan 33302, Taiwan; d000015587@cgu.edu.tw (P.-C.C.); d0101301@stmail.cgu.edu.tw (R.-C.C.); 2 Healthy Aging Research Center, Chang Gung University, Taoyuan 33302, Taiwan; 3 Neuroscience Research Center, Chang Gung Memorial Hospital, Linkou Medical Center, Taoyuan 33305, Taiwan

**Keywords:** Ca^2+^, glutamate, Na^+^, Na^+^/K^+^-ATPase, Na^+^/Ca^2+^ exchanger, mitochondria, suprachiasmatic nucleus

## Abstract

Glutamate mediates photic entrainment of the central clock in the suprachiasmatic nucleus (SCN) by evoking intracellular Ca^2+^ signaling mechanisms. However, the detailed mechanisms of glutamate-evoked Ca^2+^ signals are not entirely clear. Here, we used a ratiometric Ca^2+^ and Na^+^ imaging technique to investigate glutamate-evoked Ca^2+^ responses. The comparison of Ca^2+^ responses to glutamate (100 μM) and high (20 mM) K^+^ solution indicated slower Ca^2+^ clearance, along with rebound Ca^2+^ suppression for glutamate-evoked Ca^2+^ transients. Increasing the length of exposure time in glutamate, but not in 20 mM K^+^, slowed Ca^2+^ clearance and increased rebound Ca^2+^ suppression, a result correlated with glutamate-induced Na^+^ loads. The rebound Ca^2+^ suppression was abolished by ouabain, monensin, Na^+^-free solution, or nimodipine, suggesting an origin of activated Na^+^/K^+^-ATPase (NKA) by glutamate-induced Na^+^ loads. Ouabain or Na^+^-free solution also slowed Ca^2+^ clearance, apparently by retarding Na^+^/Ca^2+^-exchanger (NCX)-mediated Ca^2+^ extrusion. Together, our results indicated the involvement of glutamate-induced Na^+^ loads, NKA, and NCX in shaping the Ca^2+^ response to glutamate. Nevertheless, in the absence of external Na^+^ (NMDG substituted), Ca^2+^ clearance was still slower for the Ca^2+^ response to glutamate than for 20 mM K^+^, suggesting participation of additional Ca^2+^ handlers to the slower Ca^2+^ clearance under this condition.

## 1. Introduction

The suprachiasmatic nucleus (SCN) is the central clock that coordinates peripheral oscillators to control circadian rhythms in mammals [1]. Photic cues, conveying information from the retina to the SCN via the glutamatergic retinohypothalamic tract, produce biphasic phase shifts, delays at early night and advances at late night, during the dark phase of the light-dark cycle [2]. The glutamate-induced phase shifts involve Ca^2+^ entry, intracellular Ca^2+^ signaling mechanisms, gene expression, and protein synthesis [3,4]. Results from Ca^2+^ imaging and electrophysiological studies have shown that the glutamate-evoked Ca^2+^ transient is mediated by the activation of both NMDA and AMPA receptors and involves Ca^2+^ entry through the nimodipine-sensitive L-type Ca^2+^ channel [5,6,7,8,9]. The glutamate-evoked Ca^2+^ response rises rapidly in the SCN neurons in explant or punch cultures [5,6,7,9], with an EC_50_ of 3 μM [7] or 31 μM [9]. In addition, glutamate also appears to activate metabotropic glutamate receptors (mGluRs), as a type I/II mGluR agonist trans-(±)-1-amino-1,3-cyclopentanedicarboxylic acid (t-ACPD) decreases [Ca^2+^]_i_ [6] and inhibits the Ca^2+^ response to NMDA and kainate [7]. Nevertheless, the detailed mechanisms of glutamate-evoked Ca^2+^ responses are not entirely clear.

In the rat SCN cells, the Na^+^/Ca^2+^-exchanger NCX1 plays a critical role in the rapid extrusion of somatic Ca^2+^ following voltage-dependent Ca^2+^ increase [10,11]. The Na^+^/K^+^-ATPase (NKA) also plays a role in Ca^2+^ homeostasis, partly by controlling cytosolic [Na^+^] and transmembrane Na^+^ gradients to regulate NCX activity [12] (for review, see [13,14]). In view of the important role of cytosolic Na^+^ in the regulation of Ca^2+^, we hypothesized that glutamate-induced Na^+^ loads may be involved in shaping the Ca^2+^ response to glutamate. Ratiometric Ca^2+^ and Na^+^ imaging was used to determine [Ca^2+^]_i_ and [Na^+^]_i_ in cells in reduced SCN slice preparations. The Ca^2+^ response to high (20 mM) K^+^, which did not induce Na^+^ loads, was also investigated in the same cells for comparison. Our results indicate the involvement of glutamate-induced Na^+^ loads, NKA, and NCX in shaping the Ca^2+^ response to glutamate.

## 2. Results

### 2.1. Ca^2+^ Responses to 20 mM K^+^ and Glutamate

**Figure 1:**
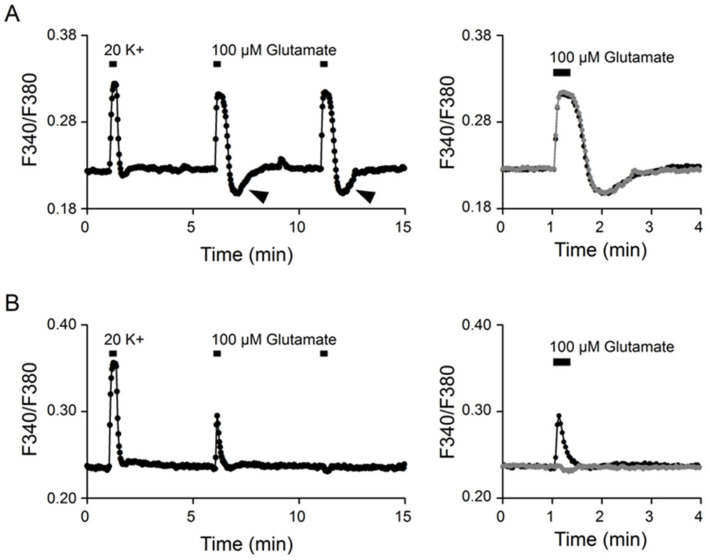
Tonic and transient Ca^2+^ responses to glutamate. (**A**) A representative experiment (an average of 15 cells) showing the tonic Ca^2+^ response to 100 μM glutamate (**left**). Note the rebound Ca^2+^ suppression (marked by arrowheads). Right panel superimposes the two consecutive glutamate responses to indicate the nearly identical kinetics, with rapid Ca^2+^ rise, to reach a plateau during the 20 s application of glutamate. (**B**) A representative experiment (an average of 17 cells) to show the transient Ca^2+^ responses to glutamate (**left**). Note the variable Ca^2+^ responses to the two consecutive application of glutamate. Right panel superimposes the two Ca^2+^ responses to indicate the totally different kinetics, one with transient Ca^2+^ increase (dark trace) and another with delayed Ca^2+^ decrease (grey trace). Note the similar kinetics of the Ca^2+^ responses to 20 mM K^+^ in the two representative experiments (**A**,**B**).

Ratiometric Ca^2+^ imaging with Fura2-AM was used to determine [Ca^2+^]_i_ in cells in reduced SCN slice preparations. Figure 1 shows two representative experiments to indicate two different types of Ca^2+^ responses to 100 μM glutamate. The experiments were performed by first applying 20 (mM) K^+^ solution for 20 s, followed by two consecutive 20 s applications of 100 μM glutamate to elicit Ca^2+^ responses (left panels). For the experiment shown in Figure 1A (an average of 15 cells), glutamate evoked a reproducible, tonic Ca^2+^ response that rapidly reached a plateau, followed by rebound Ca^2+^ suppression (marked by arrowheads) after washing out the glutamate, and the second application of glutamate 5 min later elicited an almost identical response (right panel). For the experiment shown in Figure 1B (an average of 17 cells), glutamate evoked a variable, transient Ca^2+^ response that exhibited a spike-like waveform during the 20-s application, and the second application of glutamate 5 min later failed to elicit a Ca^2+^ increase, but instead produced a small Ca^2+^ suppression (right panel). The mechanism for the variable, transient Ca^2+^ response to glutamate is not clear (see Discussion). Only the tonic glutamate responses with a rapid Ca^2+^ increase were included in the following experiments.

**Figure 2:**
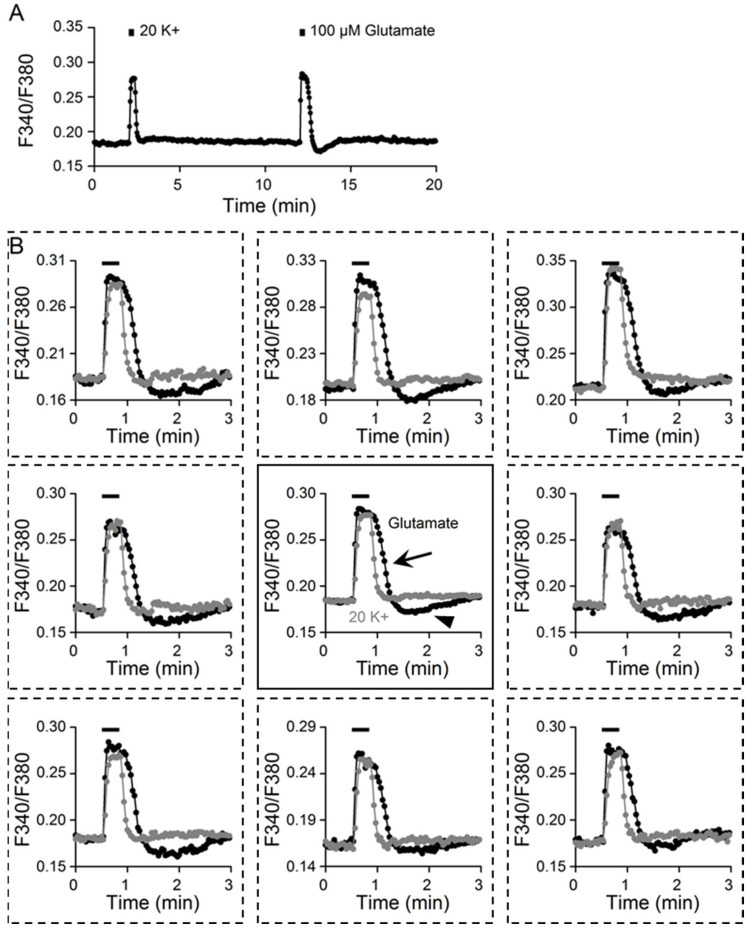
Kinetic differences between Ca^2+^ responses to 20 mM K^+^ and 100 μM glutamate. (**A**) A representative experiment showing the Ca^2+^ responses elicited by 20 mM K^+^ and 100 μM glutamate (an average of 14 cells). (**B**) Superimposition of glutamate- and 20 K^+^-evoked Ca^2+^ responses from 8 different cells (broken-lined boxes, surrounding panels). The center panel superimposed the averaged Ca^2+^ response (*n* = 14). Note the slower Ca^2+^ clearance (marked by the arrow) and rebound Ca^2+^ suppression (marked by the arrowhead) for the glutamate-evoked Ca^2+^ response.

Figure 2A shows the result from a different experiment to compare the tonic Ca^2+^ response to 20 mM K^+^ and 100 μM glutamate (an average of 14 cells). Figure 2B superimposes the Ca^2+^ response induced by 20 K^+^ and glutamate, with the center plot showing the average Ca^2+^ response (*n* = 14 cells) and the surrounding plots showing the Ca^2+^ responses from eight different SCN cells. Superimposition of the Ca^2+^ responses to glutamate (black traces) and 20 K^+^ (grey traces) revealed major differences, particularly in their Ca^2+^ clearance kinetics. While the amplitude of the Ca^2+^ increase may be comparable between the Ca^2+^ response to 20 K^+^ and 100 μM glutamate, the rate of Ca^2+^ clearance was slower (marked by the arrow), along with the occurrence of rebound Ca^2+^ suppression (marked by arrowhead), for the glutamate-evoked Ca^2+^ transient (center plot). The slower Ca^2+^ clearance and rebound Ca^2+^ suppression after glutamate washout suggest more complicated mechanisms for clearing the Ca^2+^ evoked by glutamate than by 20 K^+^-induced depolarization [10,11].

### 2.2. Na^+^ Responses to 20 mM K^+^ and Glutamate

**Figure 3:**
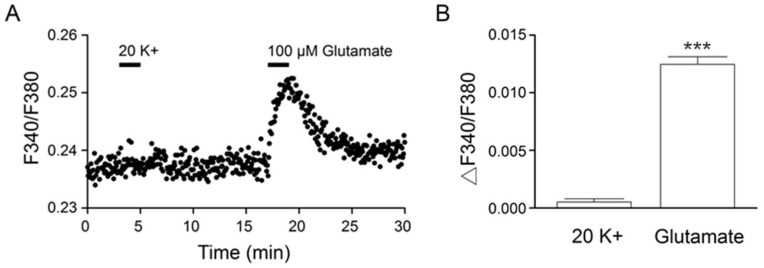
Na^2+^ responses to 20 mM K^+^ and 100 μM glutamate. (**A**) A representative experiment showing the Na^+^ responses elicited by 2 min application of 20 mM K^+^ and 100 μM glutamate (an average of 12 cells). Note the approximately linear increase in the glutamate-induced Na^+^ loads. (**B**) Statistics showing the average increase in [Na^+^]_i_ in response to 20 mM K^+^ and 100 μM glutamate. *** *p* < 0.0001.

We previously demonstrated an important role of the Na^+^/Ca^2+^-exchanger NCX1 in the rapid clearing of Ca^2+^ after high (20 and 50 mM) K^+^-evoked Ca^2+^ increases [10,11]. Furthermore, ouabain- and monensin-induced Na^+^ loads inhibit NCX activity to slow the clearance of Ca^2+^ [12]. We reasoned that the slower Ca^2+^ clearance for glutamate responses may be associated with glutamate-induced Na^+^ loads. To test this idea, we used ratiometric Na^+^ imaging to determine the effect on [Na^+^]_i_ of 20 mM K^+^ and 100 μM glutamate (Figure 3). Figure 3A shows the result obtained from one such experiment (an average of 12 cells). Indeed, 100 μM glutamate, but not 20 mM K^+^, increased [Na^+^]_i_. On average, 20 mM K^+^ and 100 μM glutamate increased [Na^+^]_i_, respectively, by 0.0005 ± 0.0002 (*n* = 181 cells from 11 experiments) and 0.0125 ± 0.0006 (*n* = 181; *p* < 0.0001; paired *t*-test) (Figure 3B).

### 2.3. Effects of Glutamate Exposure Time

**Figure 4:**
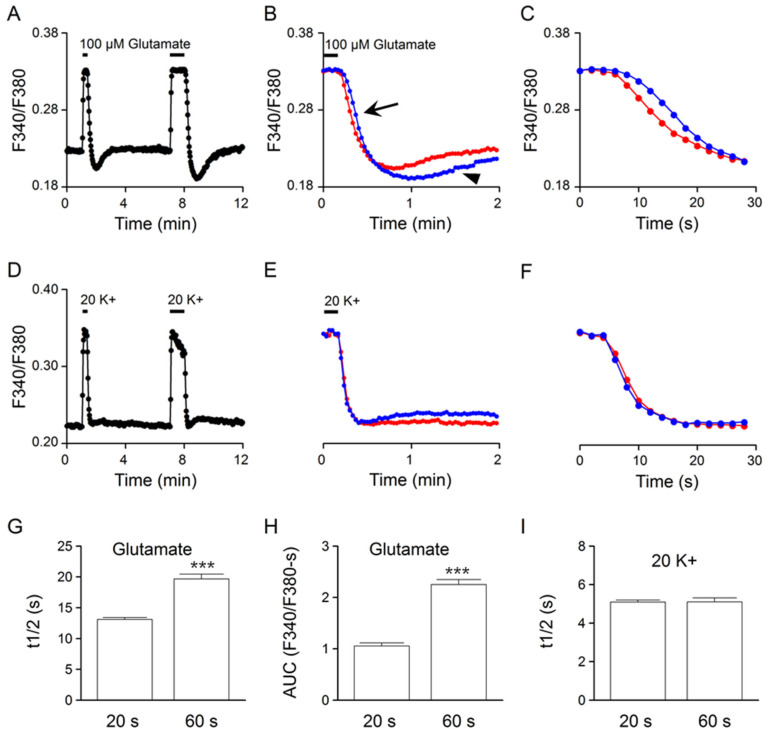
Effects on the Ca^2+^ clearance kinetics of increasing exposure time in glutamate (**A**–**C**) and in 20 mM K^+^ (**D**–**F**) from the same cells. (**A**,**D**) A representative experiment (an average of 23 cells) showing the Ca^2+^ responses to 20 and 60 s applications of 100 μM glutamate (**A**) and 20 mM K^+^ (**D**). (**B**,**E**) Superimposition of the 20 s (red traces) and 60 s (blue traces) Ca^2+^ responses at the end of 100 μM glutamate (**B**) and 20 mM K^+^ (**E**) application. Note the slower Ca^2+^ decay (marked by the arrow) and the larger and more prolonged rebound Ca^2+^ suppression (marked by the arrowhead) for the 60 s glutamate response (**B**). (**C**,**F**) Expanded time course of Ca^2+^ decay for better comparing the 20 s (red traces) and 60 s (blue traces) Ca^2+^ responses to 100 μM glutamate (**C**) and 20 mM K^+^ (**F**). (**G**,**H**) Statistics showing the effects of 20 s and 60 s glutamate application on the Ca^2+^ half-decay time (t_1/2_) (**G**) and the rebound Ca^2+^ suppression, as measured by the area under the curve (AUC) (**H**). Note the larger t_1/2_ and AUC values for the longer 60 s glutamate response. (**I**) Statistics showing a similar t_1/2_ for the Ca^2+^ responses to 20 s and 60 s 20 mM K^+^ application. *** *p* < 0.0001.

The linear rise of [Na^+^]_i_ in the first minute of glutamate application (Figure 3A) predicts a slower Ca^2+^ clearance with increasing exposure time in glutamate, if glutamate-induced Na^+^ loads were to slow the clearance of Ca^2+^. This is indeed what we have observed. Figure 4A shows the result of such an experiment to compare the Ca^2+^ response to 20- and 60-s application of 100 μM glutamate (an average of 23 cells). Superimposition of the Ca^2+^ responses at the end of glutamate application indicates a more prolonged Ca^2+^ decay (marked by the arrow) and a larger rebound Ca^2+^ suppression (marked by the arrowhead) for the 60 s response (blue trace) (Figure 4B). Figure 4C shows the expanded time course of Ca^2+^ decay to better visualize the slower clearance of Ca^2+^ for the 60 s response (blue trace). Figure 4G,H summarizes, respectively, the effect of increasing glutamate exposure time on Ca^2+^ half-decay time (t_1/2_) and rebound Ca^2+^ suppression, as measured by the area under the curve (AUC). On average, an increase in the glutamate exposure time from 20 to 60 s increased the t_1/2_ values from 13.1 ± 0.3 s (*n* = 100 cells from a total of 5 experiments) to 19.7 ± 0.8 s (*n* = 100; *p* < 0.0001; paired *t*-test) (Figure 4G) and the AUC values from 1.05 ± 0.06 (*n* = 100) to 2.25 ± 0.09 (*n* = 100; *p* < 0.0001; paired *t*-test) (Figure 4H). Altogether, the results indicate that an increase in the glutamate exposure time evoked a larger Na^+^ load (Figure 3), slower Ca^2+^ clearance (Figure 4G), and larger and more prolonged rebound Ca^2+^ suppression (Figure 4H), suggesting an important role of glutamate-induced Na^+^ loads in shaping the glutamate Ca^2+^ response kinetics.

For comparison, the same cells were also used to determine their Ca^2+^ responses to a 20 and 60 s application of 20 mM K^+^ solution (Figure 4D). Superimposition of the normalized 20 (red trace) and 60 s (blue trace) Ca^2+^ response to 20 mM K^+^ indicates nearly identical Ca^2+^ decay kinetics (Figure 4E,F). Figure 4I summarizes the effect of increasing 20 mM K^+^ exposure time on t_1/2_. On average, an increase in exposure time from 20 to 60 s changed the t_1/2_ values from 5.09 ± 0.11 s (*n* = 100) to 5.10 ± 0.21 s (*n* = 100; *p* = 0.98; paired *t*-test). The result indicates a lack of effect of increasing 20 mM K^+^ exposure time on the clearance of Ca^2+^ or the t_1/2_ value.

### 2.4. Ouabain Effects

**Figure 5:**
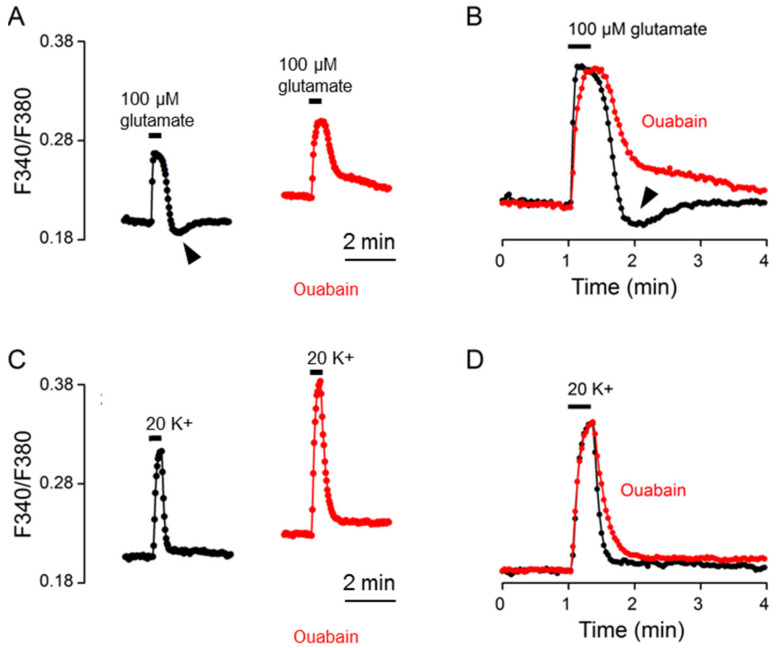
Ouabain effects on the Ca^2+^ response to 100 μM glutamate (**A**,**B**) and 20 mM K^+^ (**C**,**D**) from the same cells. (**A**,**C**) A representative experiment (an average of 13 cells) showing the effects of 10 μM ouabain on the Ca^2+^ responses to 100 μM glutamate (**A**) and 20 mM K^+^ (**C**). (**B**,**D**) Superimposition of the Ca^2+^ responses in control (black traces) and in 10 μM ouabain (red traces) elicited by 100 μM glutamate (**B**) and by 20 mM K^+^ (**D**). Ouabain slowed the clearance of Ca^2+^ for both glutamate- and 20 K^+^-evoked Ca^2+^ response. Note that ouabain eliminated the rebound Ca^2+^ suppression (marked by arrowheads) (**A**,**B**). Similar results were also obtained from 5 other experiments.

Since intracellular Na^+^ is controlled by Na^+^/K^+^-ATPase (NKA) [15] and could act on NCX to regulate [Ca^2+^]_i_ [12], we first used the cardiac glycoside ouabain (10 μM) to determine the involvement of NKA in the Ca^2+^ response to glutamate. Figure 5A shows the result obtained from one such experiment (an average of 13 cells). Of note, we previously showed that in the SCN cells, ouabain often produced a tri-phasic increase in basal [Ca^2+^]_i_, and at a concentration of 10 μM, it evoked an initial increase during the first 10~15 min, followed by a decrease to the different levels shown above, near or even below the basal level, and then an increase again to various levels close to or above the levels of initial increase (see Figure 4 and Figure 5 of [12]). For this particular experiment, the glutamate response in ouabain (red trace) was recorded at ~30 min into 10 μM ouabain when the Ca^2+^ level was higher than that before ouabain treatment (Figure 5A). The result indicates that ouabain slowed Ca^2+^ clearance and eliminated rebound Ca^2+^ suppression (marked by the arrowhead), which could be better visualized by superimposing the normalized Ca^2+^ transients in the control (black trace) and in ouabain (red trace) (Figure 5B). For comparison, the same cells were also used to determine the effect of ouabain on the Ca^2+^ response to 20 mM K^+^ (Figure 5C,D). The result indicates a larger amplitude and slower Ca^2+^ decay for 20 K^+^-evoked Ca^2+^ transients in ouabain (red trace; at ~35 min in ouabain) compared to that in the control (black trace).

The result of the ouabain-induced slowing of Ca^2+^ clearance for the 20 K^+^-evoked Ca^2+^ transient confirms our previous observation [12], and could be accounted for by the inhibition of NCX-mediated Ca^2+^ extrusion as a result of ouabain-induced Na^+^ loads. In contrast, for the glutamate-evoked Ca^2+^ transient, ouabain should additionally inhibit the extrusion of Na^+^ loaded by glutamate and thereby augment the effects of glutamate-induced Na^+^ loads on slowing Ca^2+^ clearance and eliciting rebound Ca^2+^ suppression as well. The result of ouabain-induced slower Ca^2+^ clearance is consistent with this view. In contrast, the result of the ouabain-induced elimination of rebound Ca^2+^ suppression instead suggests that glutamate-induced Na^+^ loads activate NKA to mediate rebound Ca^2+^ suppression. In other words, our results suggest that the Na^+^ loaded by glutamate shapes the Ca^2+^ response by inhibiting NCX-mediated Ca^2+^ extrusion and also activating NKA to elicit rebound Ca^2+^ suppression.

### 2.5. Monensin Effects

**Figure 6:**
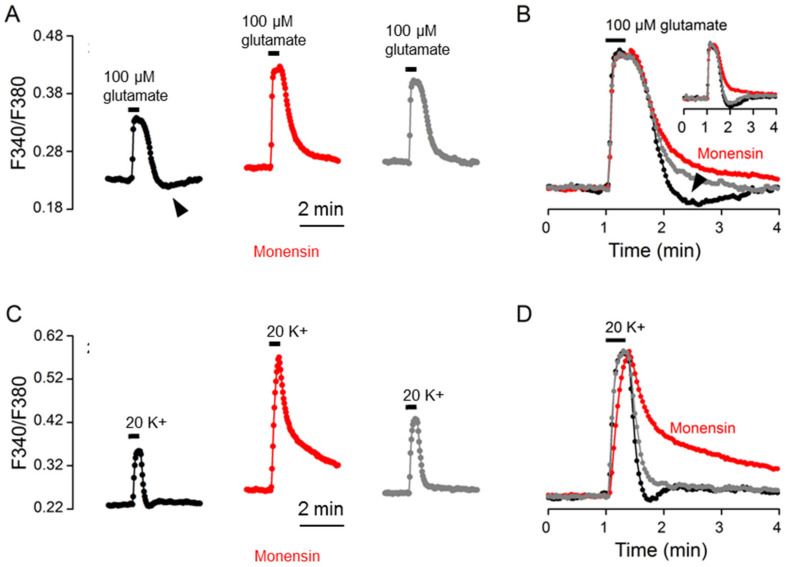
Monensin effects on the Ca^2+^ response to 100 μM glutamate (**A**,**B**) and 20 mM K^+^ (**C**,**D**) from the same cells. (**A**,**C**) A representative experiment (an average of 13 cells) showing the effects of 10 μM monensin on the Ca^2+^ responses to 100 μM glutamate (**A**) and 20 mM K^+^ (**C**). (**B**,**D**) Superimposition of the Ca^2+^ responses in the control (black traces), in 10 μM monensin (red traces), and after washout (grey traces) elicited by 100 μM glutamate (**B**) and by 20 mM K^+^ (**D**). Monensin markedly slowed the clearance of Ca^2+^ for the 20 K^+^-evoked Ca^2+^ response (**B**), but had only a minimal effect on that of glutamate-evoked Ca^2+^ response (**D**). Note that monensin eliminated the rebound Ca^2+^ suppression (marked by arrowheads) (**A**,**B**). The inset shows another experiment to indicate the reversible elimination of rebound Ca^2+^ suppression by monensin (**B**). Similar results were also obtained from 5 other experiments.

To further investigate the involvement of Na^+^ and NKA activation, we determined the effect on the Ca^2+^ response to glutamate of 10 μM monensin, which increases Na^+^ to activate NKA, hyperpolarize the resting membrane potentials [15], and slow Ca^2+^ clearance for 20 K^+^-evoked Ca^2+^ transients [12]. Figure 6A shows the result of such an experiment (an average of 13 cells). To our surprise, monensin had a minimal effect on Ca^2+^ decay and yet completely eliminated rebound Ca^2+^ suppression (marked by the arrowhead), as shown by superimposing the normalized Ca^2+^ transients in the control (black trace), monensin (red trace), and after washout (grey trace) (Figure 6B). The inset shows another experiment to indicate the reversible elimination of rebound Ca^2+^ suppression by monensin, with only a slight slowing of Ca^2+^ clearance. For comparison, the same cells were also used to determine the effect of monensin on the Ca^2+^ response to 20 mM K^+^ (Figure 6C,D). Consistent with our previous observation (Cheng et al., 2019), monensin markedly slowed the clearance of Ca^2+^ for 20 K^+^-evoked Ca^2+^ transients. For a total of 6 experiments, monensin slightly increased (*n* = 2 experiments), slightly decreased (*n* = 2), or had no effect (*n* = 2) on the t_1/2_ values for the Ca^2+^ response to glutamate, but always increased the t_1/2_ values for the Ca^2+^ response to 20 mM K^+^.

The large increase in the t_1/2_ value (or the much slowed Ca^2+^ clearance) for the Ca^2+^ response to 20 K^+^ is apparently mediated by the large Na^+^ loads induced by 10 μM monensin [12], which nevertheless failed to increase the t_1/2_ value for glutamate Ca^2+^ response and yet eliminated rebound Ca^2+^ suppression. A simple explanation is that in the presence of large Na^+^ loads induced by monensin, NKA may be maximally activated to promote the extrusion of Na^+^ loaded by glutamate and become refractory to further stimulation by glutamate-induced Na^+^ loads to elicit rebound Ca^2+^ suppression (see Discussion).

### 2.6. Nimodipine Effects

**Figure 7:**
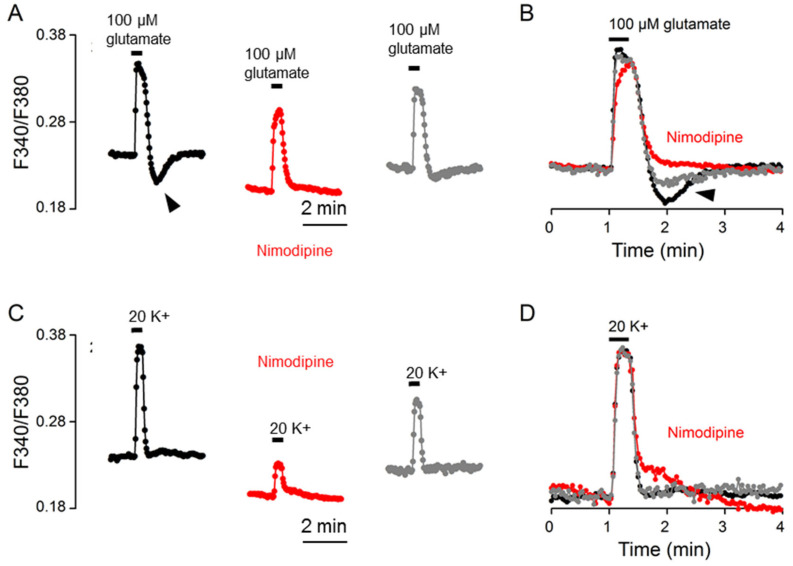
Nimodipine effects on the Ca^2+^ response to 100 μM glutamate (**A**,**B**) and 20 mM K^+^ (**C**,**D**) from the same cells. (**A**,**C**) A representative experiment (an average of 16 cells) showing the effects of 2 μM nimodipine on the Ca^2+^ responses to 100 μM glutamate (**A**) and 20 mM K^+^ (**C**). (**B**,**D**) Superimposition of the Ca^2+^ responses in the control (black traces), in 2 μM nimodipine (red traces), and after washout (grey traces) elicited by 100 μM glutamate (**B**) and by 20 mM K^+^ (**D**). Note that nimodipine eliminated rebound Ca^2+^ suppression (marked by arrowheads) (**A**,**B**), markedly reduced the amplitude of Ca^2+^ response to 20 mM K^+^ (**C**), but had no effect on Ca^2+^ half-decay time (**B**,**D**). Similar results were also obtained from 4 other experiments.

To further determine the mechanisms underlying rebound Ca^2+^ suppression, we investigated the effect of the L-type Ca^2+^ channel blocker nimodipine on the Ca^2+^ response to glutamate (Figure 7). Figure 7A shows the result of a representative experiment to indicate the effect of 2 μM nimodipine on the glutamate-evoked Ca^2+^ transient (an average of 16 cells). Figure 7B superimposes the normalized Ca^2+^ transients in the control (black trace), in nimodipine (red trace), and after washout (grey trace). Apart from lowering the basal Ca^2+^, nimodipine slowed the rate of Ca^2+^ increase to slightly reduce the amplitude, had no effect on t_1/2_, and abolished the rebound Ca^2+^ suppression (marked by the arrowhead). The result suggests that the rebound Ca^2+^ suppression is mediated by the inhibition of nimodipine-sensitive Ca^2+^ influx. For comparison, the same cells were also used to determine the effect of nimodipine on the Ca^2+^ response to 20 mM K^+^ (Figure 7C,D). Nimodipine markedly reduced the amplitude without much effect on the t_1/2_ value, confirming our previous observation [11].

### 2.7. Effects of Na^+^-Free Solution

**Figure 8:**
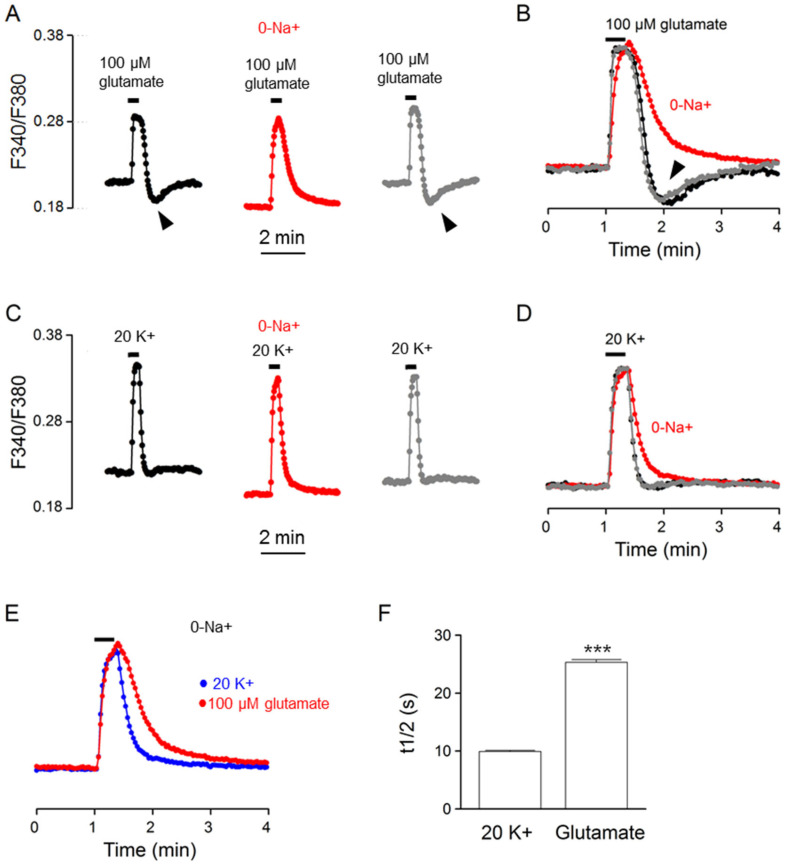
Effects of Na^+^-free solution on the Ca^2+^ response to 100 μM glutamate (**A**,**B**) and 20 mM K^+^ (**C**,**D**) from the same cells. (**A**,**C**) A representative experiment (an average of 21 cells) showing the effects of Na^+^-free solution (0-Na^+^; NMDG^+^ substituted) on the Ca^2+^ responses to 100 μM glutamate (**A**) and 20 mM K^+^ (**C**). (**B**,**D**) Superimposition of the Ca^2+^ responses in the control (black traces), in Na^+^-free solution (red traces), and after washout (grey traces) elicited by 100 μM glutamate (**B**) and by 20 mM K^+^ (**D**). Note that Na^+^-free solution eliminated rebound Ca^2+^ suppression (marked by arrowheads) (**A**,**B**) and slowed the clearance of Ca^2+^ (or increased Ca^2+^ half-decay time) (**B**,**D**). (**E**) Superimposition of the Ca^2+^ response in Na^+^-free solution to indicate a slower Ca^2+^ decay for that elicited by 100 μM glutamate (red trace) than by 20 mM K^+^ (blue trace). (**F**) Statistics showing the larger Ca^2+^ half-decay time (t_1/2_) for the Ca^2+^ response to 100 μM glutamate in Na^+^-free solution. *** *p* < 0.0001.

To determine whether NCX is indeed involved in the regulation of glutamate-evoked Ca^2+^ transients, we investigated the effect of Na^+^-free (NMDG substituted) solution on the Ca^2+^ response to glutamate. Figure 8A shows the result obtained from one such experiment (an average of 21 cells). Figure 8B superimposes the normalized Ca^2+^ transients in the control (black trace), in Na^+^-free solution (red trace), and after washout (grey trace) to indicate the reversible effect of Na^+^-free solution on slowing the Ca^2+^ decay and eliminating the rebound Ca^2+^ suppression (marked by arrowheads). For comparison, the same cells were also used to determine the effect of Na^+^-free solution on the Ca^2+^ response to 20 mM K^+^ (Figure 8C,D). Na^+^-free solution also slowed the clearance of Ca^2+^ for 20 K^+^-evoked Ca^2+^ transients, supporting our previous observation for the Ca^2+^ response to 50 mM K^+^ [10].

The slowing of Ca^2+^ clearance by Na^+^-free solution suggests the involvement of NCX in extruding Ca^2+^ for both glutamate- and 20 K^+^-evoked Ca^2+^ transients. Na^+^-free solution also eliminated the rebound Ca^2+^ suppression, suggesting an origin of glutamate-induced Na^+^ loads for the rebound Ca^2+^ suppression. Nevertheless, while Ca^2+^ clearance was slowed by the removal of external Na^+^, it is still slower for the Ca^2+^ response to glutamate, as demonstrated by comparing the normalized Ca^2+^ response to glutamate (red trace) and to 20 mM K^+^ (blue trace) (Figure 8E). On average, in the absence of external Na^+^, the t_1/2_ values for glutamate- and 20 K^+^-evoked Ca^2+^ transients averaged, respectively, 25.3 ± 0.5 s (*n* = 100 cells from 6 experiments) and 9.9 ± 0.2 s (*n* = 100; *p* < 0.0001, paired *t*-test) (Figure 8F). In other words, in the absence of NCX-mediated Ca^2+^ extrusion and glutamate-evoked Na^+^ loading, the glutamate-evoked Ca^2+^ transient still exhibits a slower rate of Ca^2+^ clearance (or larger t_1/2_). The result suggests the contribution of additional Ca^2+^ handlers to the slower Ca^2+^ clearance for glutamate response, at least under the condition of a Na^+^-free solution.

## 3. Discussion

This study demonstrates the involvement of glutamate-induced Na^+^ loads, NKA, and NCX in shaping the Ca^2+^ response to glutamate. Compared to the Ca^2+^ response to 20 mM K^+^, the glutamate-evoked Ca^2+^ response shows slower Ca^2+^ clearance, along with a rebound Ca^2+^ suppression, both being associated with glutamate-induced Na^+^ loads. The increase in intracellular Na^+^ induced by glutamate appears to inhibit NCX activity to slow Ca^2+^ extrusion and also enhance NKA activity to mediate nimodipine-sensitive rebound Ca^2+^ suppression. Nevertheless, there remain unidentified mechanisms responsible for the slower Ca^2+^ clearance for glutamate-evoked Ca^2+^ responses under the condition of occurring in a Na^+^-free solution.

### 3.1. Tonic and Transient Ca^2+^ Response to Glutamate in the SCN Cells

We show that the glutamate-evoked Ca^2+^ responses can generally be discerned into two different types: a reproducible, tonic Ca^2+^ response and a variable, transient Ca^2+^ response (Figure 1). The tonic Ca^2+^ response to glutamate rises rapidly to reach a plateau, followed by rebound Ca^2+^ suppression after glutamate washout, which is the subject of this study. While the exact mechanism remains to be determined for the variable, transient glutamate-evoked Ca^2+^ response, a previous study shows that the activation of mGluRs inhibits the Ca^2+^ response to NMDA and kainate [7]. Furthermore, the type I/II mGluR agonist t-ACPD decreases [Ca^2+^]_i_ in organotypic SCN slice cultures [6]. The variable, transient Ca^2+^ response to glutamate as reported here has not been observed in previous studies [5,6,7,9]. The reason for the discrepancy is not clear. Nevertheless, one study reports that a very small percentage (6%, 11 out of 194 cells) do not respond to 100 μM glutamate [5]. Our preliminary results suggested that the variable, transient Ca^2+^ response to glutamate appeared to involve concomitant activation of both mGluRs and ionotropic glutamate receptors (iGluRs), with the activation of the former receptors inhibiting the activation of the latter in a time-dependent manner. Further work is needed to better resolve this issue.

### 3.2. Glutamate-Induced Na^+^ Loads, NKA Activation, and NCX Inhibition

By comparing the Ca^2+^ response to 100 μM glutamate and 20 mM K^+^ solution, we found major differences in the kinetics of Ca^2+^ clearance, namely, slower Ca^2+^ clearance followed by rebound Ca^2+^ suppression for the glutamate-evoked Ca^2+^ response (Figure 2). Both the slower Ca^2+^ clearance and rebound Ca^2+^ suppression appear to be associated with glutamate-induced Na^+^ loads. First, there is a linear increase in intracellular Na^+^ during the first minute of application of 100 μM glutamate (Figure 3). Second, increasing the exposure time in glutamate, from 20 to 60 s, increases the t_1/2_ for Ca^2+^ decay (or slows the clearance of Ca^2+^) and the rebound Ca^2+^ suppression (Figure 4). In contrast, increasing the exposure time in 20 K^+^, from 20 to 60 s, had virtually no effect on the t_1/2_, a result consistent with its minimal effect on [Na^+^]_i_.

Our results further show that the Na^+^ loaded by glutamate most likely acts on both NKA and NCX to regulate the Ca^2+^ response to glutamate. On the one hand, glutamate-induced Na^+^ loads apparently enhance NKA activity to mediate the nimodipine-sensitive rebound Ca^2+^ suppression. The supporting evidence comes from the fact that the rebound Ca^2+^ suppression is eliminated by the removal of extracellular Na^+^ (NMDG substituted) (Figure 8), ouabain inhibition of NKA (Figure 5), and by the nimodipine inhibition of the L-type Ca^2+^ channels (Figure 7). Monensin also eliminates the rebound Ca^2+^ suppression (Figure 6). The result can be accounted for if NKA is already maximally activated by monensin-induced Na^+^ loads, and thus cannot be further enhanced by the Na^+^ induced by glutamate. This result is consistent with our previous findings that monensin activates NKA to hyperpolarize resting membrane potential and inhibit spontaneous firing in the SCN neurons [12,15].

On the other hand, glutamate-induced Na^+^ loads slow Ca^2+^ clearance, apparently by inhibiting NCX-mediated Ca^2+^ extrusion, an idea consistent with the result of slower Ca^2+^ decay in Na^+^-free solution to block NCX activity (Figure 8). Nevertheless, as Na^+^ loaded by glutamate activates NKA to mediate rebound Ca^2+^ suppression, the enhanced NKA activity should also promote Na^+^ extrusion to speed NCX-mediated Ca^2+^ clearance. In other words, the ability to inhibit NCX-mediated Ca^2+^ extrusion by glutamate-induced Na^+^ loading is regulated by NKA activity. This may account for the variable effects of monensin, which enhance NKA activity, on the t_1/2_ for glutamate response. In particular, for the two experiments in which monensin actually decreased t_1/2_ values (or increased Ca^2+^ clearance), it is tempting to speculate that the monensin-activated NKA may promote extrusion of Na^+^ to the extent that it actually speeds the clearance of Ca^2+^. Taken together, our results indicate a critical role of glutamate-evoked Na^+^ loads, coupled with NKA activation and NCX inhibition, to mediate slower Ca^2+^ clearance and rebound Ca^2+^ suppression for the Ca^2+^ response to glutamate.

### 3.3. Unanswered Questions

Our results also show that in the absence of external Na^+^ (NMDG^+^ substituted), the Ca^2+^ response to glutamate still has a slower Ca^2+^ clearance than 20 mM K^+^ (Figure 8). This result suggests additional unidentified mechanisms for the slower Ca^2+^ clearance for the Ca^2+^ response to glutamate, at least under the condition of occurring in Na^+^-free (NMDG^+^-substituted) solution. Further investigations are needed to answer this question. Nonetheless, is should be kept in mind that in the absence of external Na^+^ (NMDG substituted), the SCN neurons are hyperpolarized to ~–80 mV [15] and cannot fire Na^+^-dependent action potentials. As such, the Ca^2+^ handlers involved in the Ca^2+^ response elicited by glutamate might differ between the Na^+^-free solution and the physiological condition.

The detailed mechanism for the nimodipine-sensitive rebound Ca^2+^ suppression remains to be determined. The rebound Ca^2+^ suppression after glutamate washout, however, resembles rebound Ca^2+^ suppression after the washout of K^+^-free solution (see Figure 1 of [10]). We previously showed that in the rat SCN neurons, K^+^-free solution blocks NKA to induce Na^+^ loads, depolarize membrane potential, and increase firing rate, and the return to a normal K^+^ solution activates NKA to produce rebound hyperpolarization and firing inhibition [15,16]. The occurrence of rebound hyperpolarization and Ca^2+^ suppression after the washout of the K^+^-free solution has been attributed to the enhanced NKA activity as a result of Na^+^ loads induced by prior NKA blockade with K^+^-free solution [15,16]. In this context, our results suggest that while the nature of Na^+^ loads may differ between the K^+^-free solution and glutamate, the Ca^2+^ rebound suppression may share the same mechanism of enhancing NKA activity. It would be interesting to look deeper into this issue. Nevertheless, the possibility that the rebound Ca^2+^ suppression might involve the activation of ATP-sensitive K^+^ channels by the decrease in ATP levels as a result of enhanced NKA activity cannot be excluded, as has been demonstrated in the inspiratory neurons of mice [17].

### 3.4. Functional Implications

The activation of NKA by glutamate-induced Na^+^ loads may serve several functions. On the one hand, intracellular Na^+^ plays a role in regulating Ca^2+^ homeostasis in the SCN [12], and as such, glutamate-enhanced NKA activity should promote Na^+^ extrusion to regulate Ca^2+^. Our result shows that the glutamate-activated NKA actually promotes Ca^2+^ clearance to lower its level below the resting level, namely, the rebound Ca^2+^ suppression, the extent of which is proportional to glutamate-induced Na^+^ loads. In this context, NKA indeed plays a particularly important role in regulating the Ca^2+^ response to glutamate.

On the other hand, activated NKA activity has been shown to increase energy metabolism in response to electrical activity in rat posterior pituitary glands [18]. It is possible that glutamate-enhanced NKA activity may increase energy metabolism to fuel the molecular undertaking of glutamate-induced phase shifts at night. Early work has established that light increases 2-deoxyglucose uptake in the SCN in vivo at early night, when light-induced phase delays occur [19]. Similarly, the excitatory neurotransmitters glutamate, NMDA, and kainate also increase 2-deoxyglucose uptake at mid-subjective night in the SCN in vitro [20]. As protein synthesis is an energy-demanding process [21,22], and is compromised during acute energy deficiency in the brain (for review, see [23] and references herein), the light/glutamate-induced increase in glucose utilization may provide energy needed for protein synthesis. Indeed, the inhibition of protein synthesis blocks AMPA-induced early-night phase delays in the circadian firing rhythm [24]. Likewise, hypoglycemia also inhibits light-induced early-night phase delays in circadian locomotor activity [25]. The light/glutamate-induced protein synthesis appears to involve a rapid and prolonged activation of the mammalian target of rapamycin (mTOR) cascade to induce protein synthesis, in particular, in the early night [26,27].

Glutamate may also increase energy metabolism by letting Ca^2+^ enter the mitochondria to increase oxidative phosphorylation [28]. The results from our previous investigation into the effects of the mitochondrial uncoupler carbonyl cyanide-p-trifluoromethoxyphenyl hydrazone (FCCP) and Ca^2+^ channels blockers suggest mitochondrial uptake of nimodipine-insensitive Ca^2+^ component of 20 K^+^-evoked Ca^2+^ transients [11]. In this study, we show that glutamate induces a mostly nimodipine-insensitive Ca^2+^ increase (Figure 7), which could potentially enter the mitochondria to increase energy metabolism.

In conclusion, our results indicate a critical role of glutamate-induced Na^+^ loads coupled with NKA activation to regulate the Ca^2+^ response to glutamate. The glutamate-activated NKA promotes Na^+^ extrusion and mediates rebound Ca^2+^ suppression to help clear Ca^2+^ and may also serve to increase energy metabolism.

## 4. Materials and Methods

### 4.1. Hypothalamic Brain Slices and Reduced SCN Preparations

All experiments were carried out according to procedures approved by the Institutional Animal Care and Use Committee of Chang Gung University. Sprague–Dawley rats (18–24 days old) were kept in a temperature-controlled room under a 12:12 light:dark cycle (light on 0700–1900 h). Hypothalamic brain slices and reduced SCN preparations were formulated as described previously [10,11]. An animal of either sex was carefully restrained by hand to reduce stress and killed by decapitation using a small rodent guillotine without anesthesia, and the brain was put in an ice-cold artificial cerebrospinal fluid (ACSF) prebubbled with 95% O_2_-5% CO_2_. The ACSF contained (in mM): 125 NaCl, 3.5 KCl, 2 CaCl_2_, 1.5 MgCl_2_, 26 NaHCO_3_, 1.2 NaH_2_PO_4_, and 10 glucose. A coronal slice (200–300 μm) containing the SCN and the optic chiasm was cut with a DSK microslicer DTK-1000 (Ted Pella, Redding, CA, USA), and was then incubated at room temperature (22–25 °C) in the incubation solution, which contained (in mM): 140 NaCl, 3.5 KCl, 2 CaCl_2_, 1.5 MgCl_2_, 10 glucose, 10 HEPES, pH 7.4, bubbled with 100% O_2_.

For fluorescent Ca^2+^ and Na^+^ imaging, a reduced SCN preparation was obtained by excising a small piece of tissue (circa one-ninth the size of SCN) from the medial SCN using a fine needle (Cat no. 26002-10, Fine Science Tools, Foster City, CA, USA), followed by further trimming down to 4–10 smaller pieces with a short strip of razor blade. The reduced preparation (containing tens of cells, see Figure 1 of [10]) was then transferred to a coverslip precoated with poly-D-lysine (Sigma-Aldrich, St Louis, MO, USA) in a recording chamber for recording. The SCN neurons of the reduced preparation could be identified visually with an inverted microscope (Olympus IX70 and IX71, Tokyo, Japan). The preparation thus obtained allows for the rapid application of drugs [29] and has been used to demonstrate diurnal rhythms in both spontaneous firing and Na/K pump activity [30].

### 4.2. Ca^2+^ and Na^+^ Imaging in Reduced SCN Preparations

Ratiometric fluorescence imaging was carried out as described previously [10,11]. Fluorescent Ca^2+^ and Na^+^ imaging was performed, respectively, by pre-loading the SCN cells with the Ca^2+^-sensitive fluorescent indicator Fura2-acetoxymethyl ester (Fura2-AM) [31] and the Na^+^-sensitive fluorescent indicator sodium-binding benzofuran isophthalate (SBFI-AM) [32]. The reduced SCN preparation was incubated in 10 μM Fura2-AM or 15 μM SBFI-AM in 50 μL of bath solution in the dark for 60 min at 37 °C. Incubation was terminated by washing with 6 mL of bath solution, and at least 60 min was allowed for de-esterification of the dye. All imaging experiments were performed at room temperature (22–25 °C). For the experiments, the reduced SCN preparation was gently pressed on the edge against the coverslip to allow adherence of the tissue to the surface. Fluorescence signals were imaged using a charge-coupled device camera attached to an inverted microscope (Olympus IX71, Tokyo, Japan) and recorded with Xcellence 1.2 imaging software integrated with the CellIR MT20 illumination system (Olympus Biosystems, Planegg, Germany). The system used a 150-W xenon arc burner as the light source to illuminate the loaded cells. The excitation wavelengths were 340 (±12) and 380 (±14) nm, and the emitted fluorescence was collected at 510 nm. Pairs of 340/380 nm images were sampled at 0.2 Hz for Na^+^ and 0.5 Hz for Ca^2+^. Ca^2+^ and Na^+^ levels in regions of interest (ROI) over the soma were spatially averaged and presented by fluorescence ratios (F340/F380) after background subtraction. Data were analyzed and plotted with custom-made programs written in Visual Basic 6.0 and the commercial software GraphPad PRISM 8.0.1 (GraphPad Software, San Diego, CA, USA). Data were given as means ± SEM and analyzed with paired *t*-test.

### 4.3. Drugs

Stock solutions of nimodipine (20 mM in DMSO) and monensin (10 mM in 100% ethanol) were stored at –20 °C, and were diluted at least 1000 times to reach the desired final concentrations. Nimodipine was purchased from Tocris Cookson (Ellisville, MO, USA), and ouabain and monensin were obtained from Sigma-Aldrich (St Louis, MO, USA). A 20 mM K^+^ solution was prepared with equal molar substitution of K^+^ for Na^+^. Na^+^-free solutions were prepared with a total replacement of extracellular Na^+^ with N-methyl-D-glucamine (NMDG^+^). All solutions were adjusted to pH 7.4 before use.

## Data Availability

Not applicable.
